# Synthetic disulfide-bridged cyclic peptides mimic the anti-angiogenic actions of chondromodulin-I

**DOI:** 10.1111/j.1349-7006.2012.02276.x

**Published:** 2012-04-27

**Authors:** Shigenori Miura, Jun Kondo, Toru Kawakami, Chisa Shukunami, Saburo Aimoto, Hideyuki Tanaka, Yuji Hiraki

**Affiliations:** 1 Department of Cellular Differentiation, Institute for Frontier Medical SciencesKyoto University, Kyoto, Japan; 2 Research and Development Division, Science and Technology Research Center, Mitsubishi Chemical GroupKanagawa, Japan; 3 Laboratory of Protein Organic Chemistry, Institute for Protein ResearchOsaka University, Osaka, Japan

## Abstract

Chondromodulin-I (ChM-I) is a 25-kDa glycoprotein in cartilage matrix that inhibits angiogenesis. It contains two distinctive structural domains: the N-terminal third of the molecule is a hydrophilic domain that contains O-linked and N-linked oligosaccharide chains, and the C-terminal two-thirds is a hydrophobic domain that contains all of the cysteine residues. In the present study, we have attempted to further uncover the structural requirements for ChM-I to exert anti-angiogenic activity by monitoring its inhibition of the vascular endothelial growth factor (VEGF)-A-induced migration of HUVEC *in vitro*. Site-directed mutagenesis experiments revealed that the cyclic structure formed by the disulfide bridge between Cys^83^ and Cys^99^ in human ChM-I is indispensable for its anti-angiogenic function. Moreover, the C-terminal hydrophobic tail (from Trp^111^ to Val^120^) was found to play an important role in ensuring the effectiveness of ChM-I activity on HUVEC. A synthetic cyclic peptide corresponding to the ChM-I region between Ile^82^ to Arg^100^ also inhibited the migration of HUVEC, while replacing the Cys^83^ and Cys^99^ residues in this peptide with Ser completely negated this inhibitory activity. An additional synthetic cyclic peptide harboring the hydrophobic C-terminal tail of ChM-I clearly mimicked the inhibitory action of this protein on the migration of HUVEC and successfully inhibited tumor angiogenesis and growth in a xenograft mouse model of human chondrosarcoma.

Hyaline cartilage is a typical avascular tissue, and actively resists invasion by blood vessels.(1,2) However, hypertrophic/mineralized cartilage and bone are rapidly invaded by blood vessels. Devitalized hyaline cartilage can also inhibit vascular invasion, but the extraction of cartilage with guanidine hydrochloride (GuHCl) allows blood vessels to invade. These observations in earlier published studies led to the notion that a specific growth inhibitor that is extractable with GuHCl is responsible for the anti-angiogenic resistance of cartilage.^3^–^5^

We have previously found growth inhibitory activity against cultured vascular endothelial cells in GuHCl extracts from fetal bovine epiphyseal cartilage, and have identified the responsible factor to be a cartilage-specific 25-kDa glycoprotein, chondromodulin-I (ChM-I).(6) *In situ* hybridization further revealed the tissue-specific expression of ChM-I in hypovascular tissues, including hyaline cartilage, heart valves and some ocular compartments.^7^ ^8^ ^9^ ^10^ No ChM-I transcripts have been detected in hypertrophic/mineralized cartilage in the growth plate.(6,7) Hence, ChM-I is recognized as a plausible candidate for the cartilage-specific anti-angiogenic factor that had previously been envisioned.(1)

Chondromodulin-I is composed of two distinct domains (Fig. 1A).(11) The hydrophilic N-terminal domain (domain 1) contains all of the glycosylation sites in the molecule. The remainder of the molecule constitutes the hydrophobic domain (domain 2), which contains all eight cysteine residues in ChM-I and the C-terminal hydrophobic tail. Bioactivity, even if barely detectable, is evident even for the proteolytic fragment of recombinant human ChM-I (rhChM-I), which lacks most of domain 1.(11) Hence, domain 2 plays a crucial role in the anti-angiogenic activities of ChM-I.

**Fig 1:**
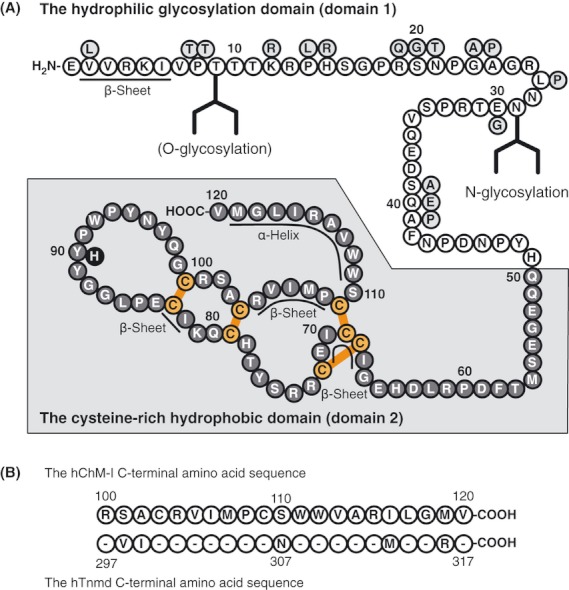
The amino acid sequence of human chondromodulin-I (hChM-I)(20). (A) Gray circles indicate amino acid residues that are not conserved in bovine ChM-I (bChM-I).^6^ ^13^ Orange bars indicate the intramolecular disulfide bonds previously determined for bChM-I.^13^ ^32^ The putative glycosylation sites are also indicated.^13^ ^32^ The locations of the α-helix and β-sheet were predicted using a new joint method based on the 3D–1D compatibility algorithm (http://mbs.cbrc.jp/papia-cgi/ssp_menu.pl). (B) The C-terminal amino acid sequences of human tenomodulin (hTnmd) and hChM-I.^17^ ^33^ The residues that are not conserved in hTnmd are indicated.

In the present study, we examined the functional role of disulfide bonds and the C-terminal hydrophobic tail of domain 2 to further map the key structural components of ChM-I through the use of recombinant mutants and synthetic mimetic peptides. Bioactivity was evaluated using a modified Boyden chamber assay of the chemotactic migration of HUVEC.(12) Finally, we examined the anti-tumor angiogenesis properties of selected ChM-I mimetic peptides in a human chondrosarcoma xenograft model *in vivo*.

## Materials and Methods

### Antibodies and reagents

Recombinant human vascular endothelial growth factor (VEGF)-A_165_ was purchased from R&D Systems (Minneapolis, MN, USA). Human vitronectin was sourced from BD Biosciences (Bedford, MA, USA). Anti-CD31 antibody and anti-collagen type II antibody were obtained from BD PharMingen (San Diego, CA, USA) and Rockland (Gilvertsville, PA, USA), respectively. Other chemicals were purchased from Sigma (St. Louis, MO, USA).

### Purification of bovine chondromodulin-I from fetal bovine epiphyseal cartilage

Bovine ChM-I (bChM-I) was purified from the 1 M GuHCl extracts of fetal bovine cartilage as described previously with some modifications.^6^ ^13^ Briefly, supernatants of cartilage extracts were dialyzed against 20 mM HEPES buffered saline (pH 7.0) with 0.5% CHAPS, and were then applied to Sepharose-4B conjugated with the anti-human ChM-I monoclonal antibody hCHM-5 (Cosmo Bio, Tokyo, Japan). The affinity column was eluted with 100 mM glycine buffer (pH 2.3). The purified bChM-I showed a single band on SDS-PAGE with 96% purity by densitometry after reverse-phase HPLC.

### Expression of recombinant human chondromodulin-I and its mutants

Recombinant human ChM-I was expressed as an N-terminal FLAG-tagged protein, as previously described.(12) The DNA fragment encoding the pre-protrypsin secretion signal (ppt)-FLAG-human ChM-I was amplified by PCR, and cloned into pCRII TOPO (Invitrogen, Carlsbad, CA, USA) to yield pCRII-ppt-FLAG-ChM-I. Constructs for ChM-I mutants were generated by PCR-based mutagenesis using pCRII-ppt-FLAG-ChM-I as a template. The primer sets used are listed in Supplementary Table S1. The DNA fragments encoding ChM-I mutants were then excised by *Not*I digestion and cloned into the pCAGGS expression vector.(14) Recombinant human ChM-I and its mutants were expressed using the FreeStyle 293 Expression system (Invitrogen) and purified with the anti-FLAG M2 affinity gel (Sigma) as described previously.(12)

### Synthesis of chondromodulin-I mimetic peptides

**Fig 2:**
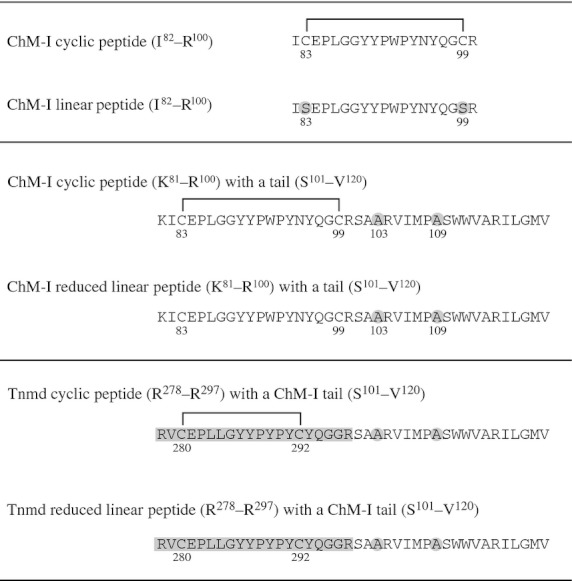
Sequences of synthetic chondromodulin-I (ChM-I) mimetic peptides. The number beneath the amino acid symbol indicates the position of the amino acid residue from the N-terminus of ChM-I or Tnmd. The cysteine residues at the position of 103 and 109 were replaced by alanine in the peptides synthesized.

All of the ChM-I mimetic peptides used in this study were synthesized by standard Fmoc solid phase peptide synthesis, and analyzed by reverse-phase HPLC and mass spectrometry (Fig. 2). ChM-I cyclic peptide is a synthetic 19-amino acid product corresponding to residues I^82^-R^100^ of hChM-I. It was cyclized through incubation at a concentration of 1 mg/mL in PBS for 30–60 min. Disulfide bond formation was confirmed by mass spectrometry. The C^83^ and C^99^ residues were replaced by serines in the ChM-I linear peptide. The ChM-I cyclic peptide with a tail is 40 amino acids in length and corresponds to the hChM-I sequence K^81^-V^120^, in which the C^103^ and C^109^ residues were substituted for alanine. The Tnmd cyclic peptide with the ChM-I tail is a chimeric peptide containing a cyclic domain of human Tnmd (R^278^–G^296^) and the C-terminal tail sequence of hChM-I with alanine substitutions at C^103^ and C^109^. The N-termini of these peptides were capped by acetylation. These products were cyclized in a mixture of hydrochloric acid and DMSO and subjected to mass spectrometry. Alternatively, the synthesized 40 amino acid peptides were kept under reducing conditions containing 1 mM DTT to prevent cyclization.

### Cell migration assay

HUVEC were grown in endothelial cell growth medium (EBM complete medium with supplements; Lonza, Walkersville, MD, USA) to subconfluence and used in the experiments at passages 4–7. The migration of HUVEC was assessed using a modified Boyden chamber assay as previously described.(12) Briefly, membrane filters (8-μm pore size, BD Biosciences) of cell culture inserts were coated with 1–2 μg/mL of vitronectin at 4°C overnight. HUVEC were serum-starved for 4 h in αMEM containing 0.5% FBS and were resuspended in αMEM containing 0.1% BSA (7 × 10^4^ cells/200 μL) after trypsinization. The cells were preincubated with test samples for 30 min, and then seeded onto the vitronectin-coated cell culture inserts. Aqueous stock solutions of ChM-I mimetic peptides were prepared at a concentration of 300 μM, and a series of dilutions were added to the cultures. In some experiments, rhChM-I was treated with 10 mM DTT at 37°C for 1 h, and added to the culture at a 200-fold dilution. Cell migration was induced by the addition of VEGF-A (20 ng/mL) in the lower chamber. After 4 h, the number of cells that had migrated to the bottom surface of the insert was counted in five representative fields per insert. These assays were performed in triplicate and repeated three times.

### Xenograft tumor model

Human chondrosarcoma OUMS-27 cells were cultured in DMEM containing 10% FBS at 37°C in 5% CO_2_.^15^ ^16^ Cell aliquots (5 × 10^6^ cells) were suspended in 0.1 mL PBS and inoculated subcutaneously into the back of 4-week-old Balb/c nu/nu mice (Shimizu Laboratory Supplies, Kyoto, Japan). When tumors reached a size of 45 mm^3^, the mice received 20 μg daily treatments of a ChM-I cyclic peptide with a tail, 5 μg rhChM-I or 50 μL PBS alone by subcutaneous injection around the tumor daily for the initial 5 days. Tumor volumes were determined as width^2^ × length × 0.52.

### Immunohistochemical analysis

Tumors were excised on day 21, fixed with 4% paraformaldehyde at 4°C overnight, embedded in the Tissue-Tek O.C.T. Compound (Sakura Finetechnical, Tokyo, Japan) and sectioned at a thickness of 8-μm. Frozen sections were washed with TBS containing 1% Tween 20 and incubated with 2% skim milk for 20 min in a humidity chamber. These sections were then incubated at 4°C overnight with a primary antibody (anti-CD31 antibody, 1:2000 dilution, or anti-type II collagen antibody, 1:800 dilution) and rinsed. For immunofluorescent analysis, the sections were incubated with Alexa Fluor 488-conjugated goat anti-rabbit IgG or Alexa Fluor 594-conjugated goat anti-rat IgG secondary antibodies (1:300 dilution, Molecular Probes, Eugene, OR, USA). The CD-31-positive areas were measured from five random fields using ImageJ (ver. 1.39u). Sections were also stained with 0.05% toluidine blue (pH 2.5).

### Statistical analysis

The significance of the differences between different groups was determined using Student's *t*-test. The differences were considered statistically significant when the *P*-value was <0.01 or 0.05.

## Results

### Inactivation of recombinant human chondromodulin-I by the reducing agent

**Fig 3:**
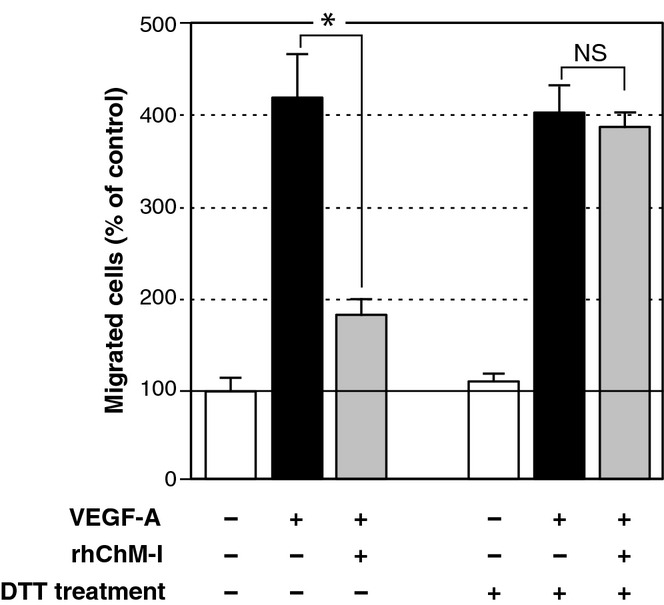
Effects of DTT on the anti-migratory activity of recombinant human chondromodulin-I (rhChM-I). Serum-starved HUVEC (7 × 10^4^ cells) were preincubated in a modified Boyden chamber for 30 min with rhChM-I (1 μg/mL), which had been incubated with or without 10 mM DTT for 1 h at 37°C, and then seeded on vitronectin-coated cell culture inserts in serum-free medium. The cells were allowed to migrate for 4 h toward vascular endothelial growth factor (VEGF)-A (20 ng/mL) added in the lower chamber. The number of cells that had migrated to the bottom surface of the insert was counted. Values are the means ± SD of a triplicate assay. The data are representative of three independent experiments with similar results. *P < 0.05; NS, not significant.

We first examined the effects of DTT on the anti-angiogenic activity of rhChM-I (Fig. 3).(12) An optimum dose of VEGF-A (20 ng/mL) stimulated the migration of HUVEC approximately fourfold over the basal level. Recombinant hChM-I (1 μg/mL, 40 nM assuming an average molecular mass of 25-kDa) successfully inhibited this VEGF-A-stimulated migration (Fig. 3). However, following incubation with DTT, rhChM-I completely lost its inhibitory activity, while DTT (50 μM) alone did not interfere with the migration of HUVEC.

### Effects of Cys to Ser mutations and a C-terminal deletion on the activity of recombinant human chondromodulin-I

**Fig 4:**
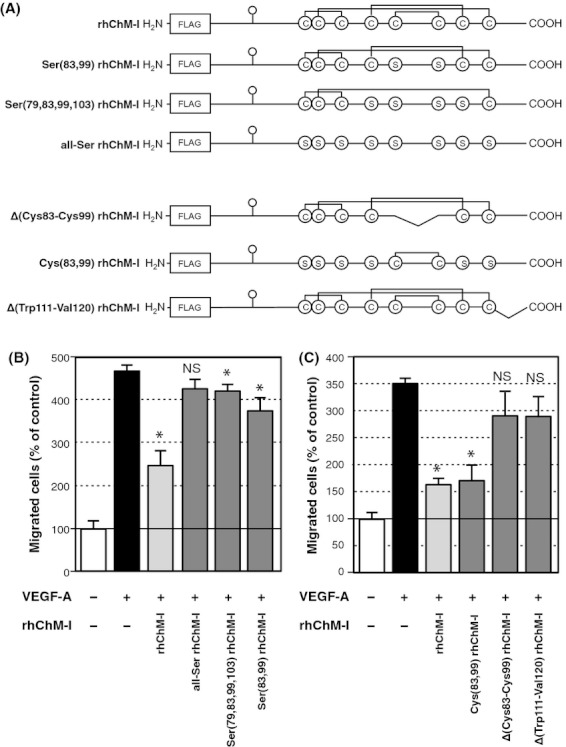
Effects of the site-directed mutagenesis of Cys residues and truncations in recombinant human chondromodulin-I (rhChM-I). (A) Schematic representation of the rhChM-I mutants generated in this study. Specific pairs of Cys residues were substituted by Ser. Δ(Cys83-Cys99) and Δ(Tryp111-Val120) rhChM-I are deletion mutants that lack the amino acids corresponding to Cys^83^–Cys^99^ and Trp^111^–Val^120^, respectively. (B,C) The anti-migratory activities of rhChM-I mutants on the vascular endothelial growth factor (VEGF)-A-induced migration of HUVEC were determined in a similar manner to Figure 2 at a fixed dose (40 nM) in culture. Values are the means ± SD of a triplicate assay and the data are representative of three independent experiments, which gave similar results. *P < 0.01 compared with control group (with VEGF-A alone); NS, not significant.

The importance of disulfide bonds to rhChM-I functions was further assessed by mutagenesis of this protein (Fig. 4A). The inhibitory action on the VEGF-A-stimulated migration was assessed by incubating these mutant proteins at a fixed dose (40 nM) in culture. The all-Ser rhChM-I mutant (1 μg/mL, 40 nM), in which all eight Cys were replaced by Ser, clearly failed to inhibit the VEGF-A-stimulated migration of HUVEC (Fig. 4B). The Ser(79,83,99,103) rhChM-I and the Ser(83,99) rhChM-I mutants were also non-inhibitory to this process. Interestingly, as shown by the Ser(83,99) rhChM-I mutant, the disruption of only one disulfide bond resulted in a significant reduction in its inhibitory activity (Fig. 4B). In agreement with this, the Cys(83,99) rhChM-I mutant, in which all of the Cys residues except for Cys^83^ and Cys^99^ were replaced by Ser, evidently inhibited the VEGF-A-stimulated migration of HUVEC (Fig. 4C). The Δ(Cys83-Cys99) rhChM-I mutant lacking the 17 amino acid residues from Cys^83^ to Cys^99^ exhibited only marginal effects, suggesting that the Cys^83^–Cys^99^ disulfide bond is important for the anti-angiogenic activity.

**Fig 5:**
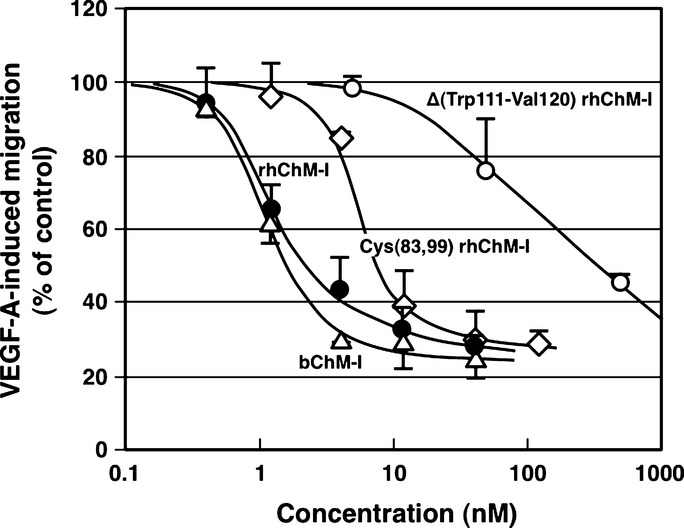
Dose-response curves for bovine chondromodulin-I (bChM-I) (△), recombinant human (rhChM-I) (●), Cys(83,99) rhChM-I (♢) and Δ(Tryp111-Val120) rhChM-I (○). The anti-migratory activities of these rhChM-I mutants determined as described for Figure 2. Values are the means ± SD of a triplicate assay and the data are representative of three independent experiments that produced similar results.

Naturally occurring bChM-I was purified from fetal bovine epiphyseal cartilage using a hCHM-5-conjugated affinity column followed by reversed-phase HPLC. The dose-response curve revealed a potent inhibitory effect of bChM-I on the VEGF-A-stimulated migration of HUVEC (ID_50_ = 1–2 nM, Fig. 5). The dose-response curve for rhChM-I was almost superimposable on that of bChM-I. The Cys(83,99) rhChM-I mutant gave a dose-response curve with a parallel slope to that of bChM-I (ID_50_ ≍ 6 nM). This parallel shift of the dose-response curve indicated that the Cys(83,99) rhChM-I mutant has an approximate fivefold weaker potency than bChM-I, but has a similar mode of action on HUVEC.

Although all eight Cys residues were maintained in the C-terminal deletion mutant, Δ(Trp111–Val120), of rhChM-I, this truncated protein showed a significant loss of activity (Fig. 4C). However, this mutant was still clearly active, albeit at a significantly higher dose compared with the wild type protein (Fig. 5). The ID_50_ value of the Δ(Trp111–Val120) mutant was approximately 150 nM from the dose-response curve, which had a significantly shallow slope and was, therefore, clearly distinct from that of the full length rhChM-I or Cys(83,99) rhChM-I.

### Bioactivity of synthetic chondromodulin-I mimetic peptides

**Fig 6:**
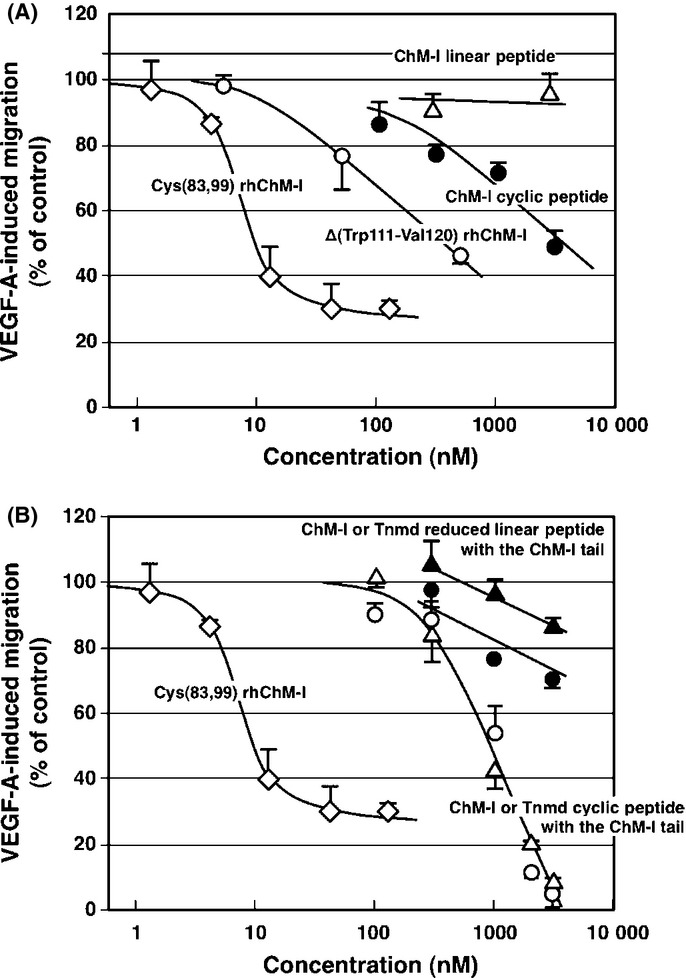
Dose-response curves for synthetic chondromodulin-I (ChM-I) mimetic peptides. (A) The anti-migratory activities of synthetic ChM-I cyclic peptide (●) or ChM-I linear peptide (△) were determined as described in Figure 2 and compared with those of the Cys(83,99) recombinant human ChM-I (rhChM-I) (♢) and Δ(Tryp111-Val120) rhChM-I (○) mutants. (B) Anti-migratory activities of a synthetic ChM-I cyclic peptide with a tail (○), ChM-I reduced linear peptide with a tail (●), Tnmd cyclic peptide with a ChM-I tail (△) or Tnmd reduced linear peptide with a ChM-I tail (▲). The anti-migratory activities of these peptides were similarly determined and compared with that of Cys(83,99) rhChM-I (♢). Values are the means ± SD of a triplicate assay and the data are representative of three independent experiments, which produced similar results.

We chemically synthesized a ChM-I cyclic peptide (I^82^–R^100^), which corresponded to the Cys^83^–Cys^99^ disulfide-bridged cyclic structure of ChM-I (Fig. 2). Owing to its hydrophobic nature, it was not possible to obtain a sufficient number of bioassay data points to prepare a complete dose-response curve, but the ChM-I cyclic peptide was clearly bioactive (Fig. 6A), with an estimated ID_50_ value of 2 μM. More importantly, the slope of the dose-response curve was particularly shallow and paralleled that of the C-terminal deletion mutant Δ(Trp111–Val120) rhChM-I. The ChM-I linear peptide, in which two Cys residues in the ChM-I cyclic peptide were replaced by Ser, did not show any appreciable activity.

We next prepared a ChM-I cyclic peptide with a C-terminal tail corresponding to the Lys^81^–Val^120^ region of the hChM-I protein (Fig. 2). The Cys^103^ and Cys^109^ residues were replaced by Ala to prevent inter-molecular and intra-molecular disulfide bonding. The addition of the C-terminal hydrophobic tail made the peptide further insoluble and, therefore, hampered the ability to perform the migration assay at higher doses (i.e. over several μM). However, this species still exhibited marked inhibitory activity in the assay (Fig. 6B). At higher doses, such as 2–3 μM, this 40-amino acid peptide inhibited cell migration almost completely. The slope of the dose-response curve became much steeper than that of the ChM-I cyclic peptide without the C-terminal tail and paralleled those of the parent rhChM-I or Cys(83,99) rhChM-I.

Tenomodulin (Tnmd), a ChM-I-related gene product, also contains a cysteine-rich domain.^17^ ^18^ ^19^ The positions of seven of the eight Cys residues in this domain are perfectly conserved, whereas the Cys^292^ position is shifted toward the N-terminal side by four amino acids, which generates the disulfide-bridged 13-membered cyclic structure of this protein.(17) As the C-terminal sequences are well conserved between ChM-I and Tnmd (Fig. 1B), we synthesized a 40-amino acid peptide (Tnmd cyclic peptide with a ChM-I tail), which contains the cyclic structure found in Tnmd (Arg^278^–Gly^296^) fused to the C-terminal sequence of ChM-I (Fig. 2). This product produced a dose-response curve superimposable with that of the tailed ChM-I cyclic peptide (Fig. 6B). This suggested that the smaller 13-membered cyclic structure in Tnmd is also functional. However, unlike the ChM-I linear peptide whose Cys residues were substituted by Ser, the ChM-I and Tnmd reduced linear peptides with the ChM-I tail prepared by DTT reduction showed only weak bioactivity (Fig. 6B).

### Inhibition of tumor angiogenesis using a chondromodulin-I mimetic peptide

**Fig 7:**
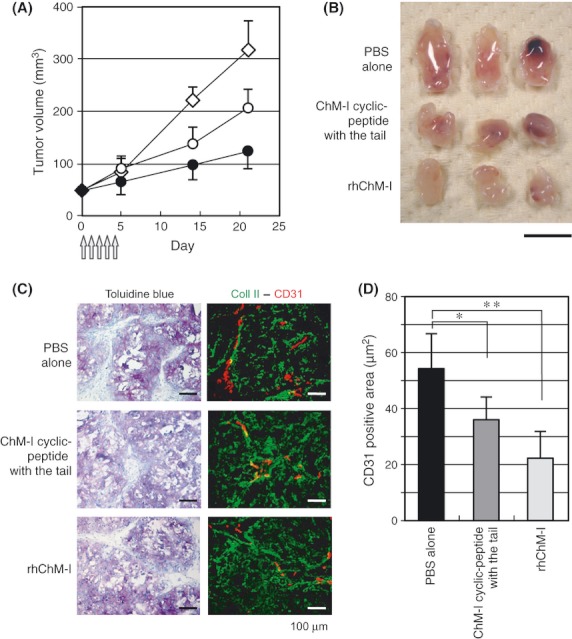
Effects of the synthetic and tailed chondromodulin-I (ChM-I) cyclic peptide on tumor angiogenesis and growth in an animal model in which OUMS-27 cells (5 × 10^6^ cells) were subcutaneously inoculated in the backs of 4 week-old nude mice. (A) Time-course of tumor volume changes. When the tumor volume reached about 45 mm^3^, each mouse was injected around the tumor mass each day for the initial 5 days with PBS (50 μL) alone (♢), PBS containing 4.3 nmol (20 μg) ChM-I cyclic peptide with a tail (○), or PBS containing 0.2 nmol (5 μg) recombinant human ChM-I (rhChM-I) (●) (arrows). The tumor volumes were determined by the width^2^ × length × 0.52. Values are the means ± SD from at least six animals per group. (B) Gross appearance of tumors excised on day 21. Bar, 10 mm. (C) Immunohistochemical staining of the CD31-positive vasculature. On day 21, tumor tissues were excised, fixed, and cross-sectioned. The sections were then stained with toluidine blue (left panels), and semi-serial sections were stained with an anti-type II collagen antibody (*green* signal in the right panels) and an anti-CD31 antibody (*red* signal in the right panels), respectively. Bars, 100 μm. (D) The CD31-positive area was measured as described in the methods section. Values are the means ± SD of five tumors per group. *P < 0.05, **P < 0.01.

When subcutaneously transplanted into nude mice, human OUMS-27 chondrosarcoma cells actively produce a cartilage-like matrix, including type II collagen and aggrecan, but have no capacity to produce ChM-I.(15) Instead, the cells secrete VEGF-A_165_ into the culture medium.(16) Thus, the cells form a vascularized but translucent tumor mass with cartilage-like ECM in nude mice. Using this xenograft tumor model, we examined the effects of a ChM-I mimetic peptide on tumor angiogenesis *in vivo*. The OUMS-27 chondrosarcoma that developed in these animals was treated with PBS daily for the first 5 consecutive days, with the volume rapidly increased from 45 mm^3^ to approximately 300 mm^3^ by 3 weeks (Fig. 7A). In contrast, the tumors in this model that received rhChM-I (5 μg each for the initial 5 days) clearly grew slowly to reach a size of approximately 120 mm^3^. ChM-I cyclic peptide with a tail (20 μg each for the initial 5 days) also clearly inhibited tumor growth (Fig. 7A). The invasion of blood vessels could be readily observed through the translucent tumor tissues (Fig. 7B) and the CD31-positive vasculature seen in the type II collagen-positive cartilage-like tumor ECM (Fig. 7C). As shown in Figure 7(D), tumor angiogenesis was clearly inhibited by the tailed ChM-I cyclic peptide.

## Discussion

Chondromodulin-I and Tnmd form an anti-angiogenic protein family characterized by a unique disulfide-bridged hydrophobic domain at their C-termini.^17^ ^20^ ^21^ This structure is implicated in the heat-stable and reduction-sensitive nature of bChM-I^6^ ^13^. In fact, incubation of rhChM-I with DTT completely abolishes its activity against the VEGF-A-stimulated migration of HUVEC. The Cys(83,99) rhChM-I mutant in our current study successfully inhibited VEGF-A-stimulated migration of HUVEC. Deletion of the 17-amino-acid stretch from Cys^83^ to Cys^99^ resulted in the loss of this inhibitory activity. Synthetic ChM-I mimetic peptides revealed that even the disulfide bridge-closed cyclic structure alone could be inhibitory to this migratory event. Thus, this disulfide bridge-closed cyclic structure might represent a core structure that underlies the activity of the ChM-I/Tnmd family of proteins.

Interestingly, the disulfide bridge-closed cyclic moiety is rich in aromatic residues, such as Tyr and Trp. We speculate that this particular cyclic structure contributes to downstream signaling by presenting these residues to the putative cognate receptor(s), as exemplified by the single disulfide bridge-containing peptide mimetics that interfere with ErbB receptor signaling by presenting specific amino acid residues onto the ligand recognition surface of the receptor.^22^ ^23^ Cryptic angiogenesis inhibitors derived from vascular basement membrane collagens, such as endostatin and tumstatin, also exhibit anti-migratory and anti-proliferative properties against cultured vascular endothelial cells.^24^ ^25^ The disulfide-bridge closed cyclic peptide, which corresponds to the C-terminal region of endostatin (Fragment IV), was found to be an inhibitor of endothelial cell migration and proliferation.(26) However, a non-cyclic N-terminal peptide (Fragment I) that contains an α-helix also had an inhibitory effect on the migration of HUVEC and angiogenesis *in vivo*.^27^ ^28^ In the case of tumstatin, the anti-angiogenic activity is not dependent on its disulfide-bridged structure.^29^ ^30^ Unlike these cryptic inhibitors,(31) function-blocking antibodies against β1 and αvβ3 integrins did not interfere with the inhibitory action of ChM-I,(12) suggesting that ChM-I carries a unique anti-angiogenic sequence motif and acts on endothelial cells through a mechanism distinct from these cryptic angiogenesis inhibitors.

The conserved hydrophobic C-terminal tail is also characteristic of ChM-I and Tnmd.(17) Previous measurements of far-UV circular dichroic spectra have suggested that the bioactive glycosylated rhChM-I contains an α-helix.(11) The primary sequence of ChM-I indicates that an α-helix is predicted to localize at its hydrophobic C-terminus. Deletion of these C-terminal residues renders rhChM-I significantly less sensitive to the dosage. The dose-response curve of this truncated rhChM-I mutant was found to be parallel to that of the ChM-I cyclic peptide. Addition of the ChM-I C-terminal tail to the ChM-I cyclic peptide resulted in a restoration of the normal dosage-sensitive response. Hence, we speculate that the hydrophobic tail (Trp^110^–Val^120^) of ChM-I may assist with its signaling by facilitating the presentation of its disulfide-bridged core structure (Cys^83^–Cys^99^) to the putative ChM-I receptor through the binding to a low-affinity site or co-receptor.

A ChM-I cyclic peptide conjugated directly to the C-terminal tail (Trp^111^–Val^120^) of ChM-I and a ChM-I cyclic peptide with a tail having the polyethylene glycol moiety (NH-CH_2_CH_2_O-CH_2_CH_2_O-CH_2_CO)_3_ in place of the intervening nine residues (Ala^102^-Ser^110^) were synthesized. Both of these peptides were substantially insoluble in culture medium where they formed aggregates and this prevented an examination of their bioactivity *in vitro* (data not shown). The results of MALDI-TOF-MS analysis suggests that a substantial portion (30–50%) of these peptides is spontaneously oxidized at the C-terminal Met^119^ residue. These observations led us to speculate that the disulfide-bridged cyclic structure and C-terminal tail have to be adequately separated from each other to function appropriately. Therefore, the intervening nine amino acids may contribute to the separation of these functional units, without which the C-terminal hydrophobic tail cannot be internally folded. In this regard, the parent ChM-I molecule has a disulfide-bridged cyclic structure (Cys^83^-Cys^99^) that is effectively separated from the hydrophobic C-terminal tail by anti-parallel stretches (Cys^68^–Cys^83^ and Cys^99^–Cys^109^) interconnected with disulfide bonds.

In summary, we have in our present study dissected out the structural elements of ChM-I that are functionally important for its anti-angiogenic properties. The design and application of bioactive synthetic ChM-I mimetic peptides will also help with the identification of ChM-I/Tnmd receptors and may contribute to the development of anti-angiogenic therapies.

## Supporting Information

**Table S1.** Primer sets used in the mutagenesis experiments. Please note: Wiley-Blackwell are not responsible for the content or functionality of any supporting materials supplied by the authors. Any queries (other than missing material) should be directed to the corresponding author for the article.
