# Supplementary material for: Synthetic disulfide-bridged cyclic peptides mimic the anti-angiogenic actions of chondromodulin-I
Source: Cancer Sci. 2012 Apr 27;103(7):1311–8. doi: 10.1111/j.1349-7006.2012.02276.x (PMC3492907; doi:10.1111/j.1349-7006.2012.02276.x)
Supplement: Supplementary file 1 — Table S1. Primer sets used in the mutagenesis experiments. [file cas0103-1311-SD1.pdf]

Table S1. Primer sets used in the mutagenesis

**Mutant proteins***	5'-Forward primer-3'
Template plasmids	5'-Reverse primer-3'
Ser(83) rhChM-I	ACCCACTGCCAGAAGATCAG
pCRII-ppt-FLAG-ChM-I	GTAGCTCCGCCTACATTCTATA
**Ser(83, 99) rhChM-I**	CGTTCGGCCTGCAGAGTCAT
pCRII-ppt-FLAG-Ser (83)	GCTGCCTTGATAATTATAAGGC
Ser(79, 83, 99) rhChM-I	ACCCACAGCCAGAAGATCAG
pCRII-ppt-FLAG-Ser (83, 99)	GTAGCTCCGCCTACATTCTATA
**Ser(79, 83, 99, 103) rhChM-I**	CGTTCGGCCAGCAGAGTCAT
pCRII-ppt-FLAG-Ser (79, 83, 99)	GCTGCCTTGATAATTATAAGGC
Ser(68, 69, 72, 79, 83, 99, 103) rhChM-I	AGCTACACCCACAGCCAGAAG
pCRII-ppt-FLAG-Ser (79, 83, 99, 103)	CCGCCTACTTTCTATACTACTG
**all-Ser rhChM-I**	CATGCCAAGTAGCTGGTGGG
pCRII-ppt-FLAG-Ser (68, 69, 72, 79, 83, 99, 103)	ATGACTCTGCTGGCCGAACG
Ser(68, 69, 72, 79) rhChM-I	AGCTACACCCACAGCCAGAAG
pCRII-ppt-FLAG-ChM-I	CCGCCTACTTTCTATACTACTG
**Cys(83, 99) rhChM-I**	CATGCCAAGTAGCTGGTGGG
pCRII-ppt-FLAG-Ser (68, 69, 72, 79)	ATGACTCTGCTGGCCGAACG
**Δ(Cys83-Cys99) rhChM-I**	CGTTCGGCCTGCAGAGTCAT
pCRII-ppt-FLAG-ChM-I	GATCTTCTGGCAGTGGGTG
**Δ(Trp111-Val120) rhChM-I**	GCGGCCGCTAATTAGTTAGTC
pCRII-ppt-FLAG-ChM-I	TCAGCTACATGGCATGATGAC

*Schematic representations of these mutant ChM-I proteins are shown in Fig. 3A.
